# Observations on the Manner in Which Caloric Is Distributed and Retained on the Surface of the Human Body

**Published:** 1817-04

**Authors:** M. Bres


*For the London Medical and Physical Journal.*

### *Observations on the Manner in which Caloric is distributed and retained on the Surface of the Human Body*; by M. Bres.

THE heat we are susceptible of feeling may be divided into three species, according to the causes which produce it.

The first is heat exerted on the body by the air and external bodies, whatever may be the cause.

The second is the individual heat which seems to have its permanent source in the organs themselves, whatever be its origin.

The third is the heat which is developed by the friction of divers parts of the body against each other.

To study these three kinds of caloric, in their relations with the animal organization, it would be proper to study heat in its primitive species—1, as produced by the sun; 2, by artificial fire; 3, by rubbing external bodies against the individual; 4, by vital foreign heat; 5, by individual heat received by the individual himself; 6, by fermentation and the action of gases.

Individual heat ought to be studied—1, in the pulmonary organs, which, if they be not the source, seem to be the seat; 2, in the other organs, considering them in the trunk, the head, in the abdomen, in the thoracic and abdominal extremities, &c.; 3, in inflammations, and different pathological states.

Heat of the third species is to be studied in the action of the different parts on each other, and in their friction.

After having studied the action of heat on this plan, it would be necessary to make observations on the means employed by nature to preserve vital heat, and to endow the human body with the faculty of struggling with success against the caprices of the atmosphere.

I shall here confine myself to the outline of a more important labour, and inquire only how heat is distributed on the surface of the human body, and especially how heat is

 retained retained in the places to which the organic powers have directed it.

Who is ignorant that grease or fat, by its quality as a non-conductor of heat, concentrates heat in the individual, in surrounding it with an almost general layer? yet the animal fat, by means of pores, which open and close as necessity requires, permits the caloric a sufficient circulation to establish between the individual or animal heat, and external heat, that sort of equilibrium from which results the state of what we call *feeling comfortable.*

Who is ignorant that the thorax is the part of the body where the caloric seems to develope itself with the greatest energy? In man, it is in the largest cavity, it is from this seat that heat seems to escape in rays through all the parts of the body.

Heat, in escaping from the centre to the circumference, finds places where it establishes a kind of reservoirs or seats.

To establish these seats of caloric on the surface of the body, the organic powers have employed the approximation of the parts; in fact, the parts approaching each other unite their caloric, and prevent the action of the ambient air upon it, which would destroy it.

Thus, the arm-pits become two seats of caloric, by the contact of the arm on the thorax. These two seats are the more useful, as they are placed near the surface of the most massive part of the body, and which, on this account, requires the greater quantity of heat. These two seats, placed between the anterior and posterior parts of the thorax, are the most favourable places for the uniform distribution of caloric.

The various flexions of the thoracic members are also made in the points which become new seats of heat, which may be examined in their turn, in the bending of the elbow, and the flexion of various parts of the palm of the hand and fingers.

The thoracic members, by the various positions which they are susceptible of taking, can augment or diminish the sensible heat. Cold leads us to bend the articulations of these members, and to bring them to the trunk, and to reduce our body into the least volume possible, in order that the vital heat, more concentrated, may preserve all its energy. We know what advantage it is to close the hand, to avoid the cold which would seize the fingers: the number of these, and the pieces that compose them, permit the establishment of a certain number of these seats, the more useful, as they are found on the smallest organs, and the farthest removed from the general centre of heat.

The

The head has a considerable seat of caloric under the neck, of which the effect is augmented in bending it towards the sternum. It also possesses very useful seats behind the ears, and under the hair.

But the most important fact presented by the head, under the relation of caloric, is the art of breathing. In fact, when, in breathing, a vapour, charged with caloric, escapes from the mouth and nose, and spreads, particularly in exhaling from the nose, in such a manner as to direct itself on the general centre of heat, on the thorax, then the internal heat seems to throw off its superfluity, and employ it to protect the exterior parts.

Amongst the numerous advantages which man finds in hanging the head in cold weather, we may remark that of directing the heat exhaled by the lungs on the exterior of the breast, and protecting, by the warmth, the seat of heat itself.

When we walk quickly, the air, which leaves the lungs charged with caloric, is guided by the atmospheric air, and no longer descends upon the sternum. It directs itself only on the neck, where, perhaps, it becomes of great utility in preventing accidents.

On examining the abdominal extremities, we shall discover several seats of heat in the various articulations; the most important is that which is found between the two thighs. These parts, in approximating their caloric, preserve it; and their considerable volume serves to prolong the duration of its existence. The muscles of these parts tend sometimes to augment their heat by their reciprocal friction: this heat is rendered still more considerable by the flexion of the femur on the abdomen; and we ought, perhaps, to remark here, that the gas which escapes from the anus throws a new heat on those parts, and has some relation, under this point of view, with the air which exhales from the lungs in the act of breathing.

The flexion of the leg upon the thigh establishes a centre of heat at a considerable distance from the general centre of caloric; and the ham, in bending, becomes very useful for the preservation of the caloric, which we obtain in a still more positive manner in putting one thigh upon the other.

The same advantage is found in the flexion of the toes, and in their approximation, each of which is more considerable than in the hand, and which was necessary, on account of their greater distance from the general centre of heat.

We

We must here remark a very important fact—it is that the flexion of all the members tend to throw upon the abdomen, the principal seat of external heat; the general flexion of the trunk itself concurs to this end.

When the atmosphere is cold, all our members tend to approach the abdomen; the vertebral column bends forward, and the head bows over the breast. Thus are concentrated the seats of caloric, to augment their energy; and thus all these seats seem to unite to form only one.

After having observed this *concensus* of the parts, to fix the centre of external heat on the anterior part of the trunk, ought we to be led to conclude the necessity of an external heat, active and permanent, brought to this part? or rather ought we simply to consider this *concensus* as necessary to fix the heat in a general centre, where the approximation of so many parts tend to preserve it?

There is no doubt that caloric is one of the agents of digestion; it is also necessary to gestation. These two phenomena, which we may regard as the most important in our organization, have their seat in the space where heat is the most easily concentrated, and the longest preserved.

During sleep, caloric has a permanent energetic action on the organization. The suffering the predominance of the flexors over the extensors to manifest itself, permits that general state of flexion, the effects of which we have observed, and which concentrates the heat on the anterior part of the trunk. It is then that the caloric is divided between the parts. All the members, in bending on the trunk, go, if we may so express ourselves, to seek it at its source; and, in impregnating themselves with caloric, assume that state of dilation so necessary to the distribution of the nourishment in the parts.

The form which the body assumes in this state is, that of the child in the bosom of its mother; this form, so well adapted to that of the matrix, is a long time that of the body of the infant, to which heat is so necessary in the first months of its existence. This form is also that of animals which sleep the greater part of winter, and which, deprived of the motion of the members, and of the motion of nutritive substances, in the interior of the body, have need of preserving their heat by every possible means.

Setting huddled up *(accroupissement)* is a state much more habitual amongst the northern nations than amongst those of the south, on account of the atmospheric cold; and, perhaps, one might refer to this habit the difference between the people of the north and those of the south in their their carriage. In general, the southern nations walk erect, while those of the north have their bodies more or less bent. It would be carrying the spirit of system too far to suppose, that the deformity in the figure of the Laplanders depends partly on their custom of setting squat during a part of their existence.

Every moment devoted to the examination of the admirable economy of the human body, enables us to discover in it new perfections.

I will here pause, inviting the reader to reflect on the agreeable sensations, that the faculty of diminishing or augmenting his external sensible heat may procure him by simply approximating or separating the parts from each other. Is not this advantage, for many individuals, one of the benefits of the creating genius which we enjoy without appreciating it? Happy, if, by these observations, I may call forth a new homage of admiration of that organizing Power which has made nothing without an object, and whose object has only been the multiplication of our agreeable sensations.

---

*For the London Medical and Physical Journal.*

